# Measuring broader wellbeing in mental health services: validity of the German language OxCAP-MH capability instrument

**DOI:** 10.1007/s11136-019-02187-9

**Published:** 2019-04-27

**Authors:** Agata Łaszewska, Markus Schwab, Eva Leutner, Marold Oberrauter, Georg Spiel, Judit Simon

**Affiliations:** 1 Department of Health Economics, Center for Public Health, Medical University of Vienna, Kinderspitalgasse 15/1, 1090 Vienna, Austria; 2 pro mente Forschung, Villacher Straße 161, 9020 Klagenfurt am Wörthersee, Austria; 3 pro mente kärnten GmbH, Villacher Straße 161, 9020 Klagenfurt am Wörthersee, Austria; 4 Department of Psychiatry, Warneford Hospital, University of Oxford, Warneford Ln, Oxford, OX3 7JX UK; 5 HERC, Nuffield Department of Population Health, University of Oxford, Richard Doll Building, Old Road Campus, Oxford, OX3 7LF UK

**Keywords:** Quality of life, PROM, Capabilities, Psychometric validation, Mental health, Wellbeing

## Abstract

**Purpose:**

The OxCAP-MH capabilities questionnaire was developed and validated in the UK for outcome measurement in mental health clinical studies. Its broader wellbeing construct or validity in routine mental health services so far has not been assessed. The objectives were to investigate the extent the OxCAP-MH measures broader wellbeing compared to the EQ-5D-5L and to test psychometric properties of the German language OxCAP-MH in routine mental health services in Austria.

**Methods:**

Study sample consisted of patients in socio-psychiatric services (*n* = 159) assessed at baseline and 6-month follow-up. Underlying factors associated with quality-of-life/wellbeing concepts measured by the OxCAP-MH and EQ-5D-5L were identified in exploratory factor analysis (EFA). Responsiveness was assessed using anchor questionnaires and standardised response mean (SRM). For discriminant validity, subgroups of respondents were compared using *t* test and one-way ANOVA. Test–retest analysis was assessed for a period of maximum 30 days from the baseline assessment with intra-class correlation coefficient (ICC).

**Results:**

EFA identified a two-factor structure. All EQ-5D-5L items and seven OxCAP-MH items loaded on one factor and nine remaining OxCAP-MH items loaded on a separate factor. Responsiveness was found for patients who improved in anchor questionnaire scores with large or moderate SRM statistics. OxCAP-MH discriminated between various groups in univariable and multivariable analyses. Reliability of the German language OxCAP-MH was confirmed by ICC of 0.80.

**Conclusions:**

Besides providing evidence that the OxCAP-MH measures broader wellbeing constructs beyond traditional health-related quality of life, the study also confirms the validity of the instrument for implementation in routine evaluation of mental health services.

**Electronic supplementary material:**

The online version of this article (10.1007/s11136-019-02187-9) contains supplementary material, which is available to authorized users.

## Introduction

There has been a general debate whether the currently used generic health-related quality-of-life (HRQoL) measures, for example, EQ-5D commonly used for quality-adjusted life years (QALYs) calculations, sufficiently reflect the most important dimensions of patients’ wellbeing for evaluative purposes [1–3]. Some authors argue that an alternative way of assessment should be implemented to capture both health and non-health effects of evaluated interventions especially in evaluations conducted from a societal perspective [4–8]. Moving beyond the QALYs framework to account for non-health outcomes in economic evaluations requires the use of measures that capture a broader set of patient outcomes [4, 9]. This is particularly relevant for mental health interventions which are expected not only to improve health outcomes, but also impact areas of recovery including social relationships, hope and optimism about the future, identity, meaning of life and empowerment of personal autonomy [10].

The capabilities framework has gained attention as an alternative to the traditional welfare economics approach used in evaluating quality of life. The term ‘capability’ in the context of social equality or inequality was defined by Amartya Sen in the field of the development economics research [11]. It meant to indicate a space of each individual to do things that he or she chooses and values in life [12].

A recent literature review by Mittchel et al. demonstrated increasing interest in the application of the capability approach in the field of health [13]. The review pointed out that several new instruments have been developed in the last decade which applied the capability approach in the assessment of health and social care interventions. These instruments demonstrated good psychometric properties in different disease areas, population groups and settings [14–19]. Moreover, studies showed that the capability-based instruments seem to measure broader aspects of human’s wellbeing compared to, for example, EQ-5D [8, 20, 21]. There is a growing body of literature about the use of the capability-based instruments in economic evaluations to support decisions on allocation of resources [22–24]. Recently, the National Institute for Health and Care Excellence (NICE) in the UK recommended the use of ASCOT and ICECAP-O capability questionnaires in economic evaluations of social care interventions [25].

Sen’s capabilities theory was further developed by Martha Nussbaum who proposed a list of 10 central human capabilities [11] which served as a basis for the OxCAP-MH (Oxford CAPabilities questionnaire-Mental Health) questionnaire (Online Supplement Table 1). The OxCAP-MH was originally designed to measure capabilities in patients with mental disorders in the UK [7] and has been successfully deployed in several trial-based mental health economic evaluations since [26–28]. Recently, OxCAP-MH was translated to German to facilitate its use in German-speaking countries [29].

The original English version of the OxCAP-MH was tested in a randomised controlled trial on a sample of patients with a primary diagnosis of psychotic disorders [7, 14]. Findings from Vergunst et al. demonstrated good feasibility of the questionnaire in this patient group as well as its good psychometric properties in terms of reliability and validity [14, 30]. However, the dimensionality of the OxCAP-MH attributes using exploratory factor analysis (EFA) and more elaborate comparison to the EQ-5D in terms of the measured quality-of-life and broader wellbeing constructs have not yet been investigated. Furthermore, the questionnaire has not been tested in terms of its responsiveness and discriminant validity. Also the use of this instrument has not been formally tested in a routine health services evaluation setting.

Therefore, the objectives of this study were (1) to examine the dimensionality of the OxCAP-MH and to analyse to what extent OxCAP-MH measures broader concept of wellbeing compared to the EQ-5D-5L, (2) to test the psychometric validity of the German language instrument in routine mental health services and (3) to compare the results to those of the original UK psychometric validation.

## Methods

### Study participants and procedure

Study participants were approached by their formal carers in the routine setting of socio-psychiatric services, organised by pro mente kärnten, in the Austrian state of Carinthia. Out of nine states in Austria, Carinthia is sixth in terms of the size of the population which constitutes around 6.4% of Austrian general population [31]. It has GDP per capita of 34,300 EUR and the second highest unemployment rate in Austria (10.2%) [31, 32]. Pro mente kärnten is the biggest non-profit community-based organisation located in Carinthia that offers comprehensive support and treatment for mentally ill adults.

The eligibility criteria included the following: patients with diagnosed mental disorder receiving socio-psychiatric services, ability and willingness to give a written consent, age above 18, fluent in German and not in active phase of the disease.

The study was aligned with the clinical routine assessment system implemented in the socio-psychiatric services which is used to collect patient information every 6 months. At baseline, patients completed self-reported questionnaires during their regularly scheduled meetings with their carers. As routine follow-up, the same self-reported questionnaires were filled out at 6-month intervals. Other routinely collected data included socio-demographic characteristics, diagnostic codes according to the Diagnostic and Statistical Manual of Mental Disorders (DSM-IV) [33] and observer-rated questionnaires filled out by the carers (Global Assessment of Functioning (GAF) and Mini-ICF-APP Social Functioning Scale).

In addition to the routinely collected information, study participants were asked to complete the OxCAP-MH and EQ-5D-5L at baseline and at 6-month follow-up. For the test–retest analysis, the OxCAP-MH was completed once at the next scheduled care meeting within 30 days of the baseline assessment.

The study had been approved by the Ethics Committee of the Medical University of Vienna (EK-Nr: 2191/2015, Votum: 08.03.2016).

### Instruments

Instruments included German versions of the World Health Organization Quality of Life BREF (WHOQOL-BREF), the Short Brief Symptom Inventory-18 (BSI-18), Global Assessment of Functioning (GAF), Mini-ICF-APP, EQ-5D-5L and OxCAP-MH.

WHOQOL-BREF is a generic self-reported tool that covers four dimensions (physical, psychological, social relationships and environment) plus overall value for quality of life and satisfaction with individual’s health. It consists of a total of 26 items with five levels of answers. Higher scores ranging from 0 to 100 denote higher quality of life. The instrument is widely used in international research projects [34]. The Cronbach’s alpha for WHOQOL-BREF in a German sample of respondents from the general population and somatic and psychiatric patients was 0.88, 0.83, 0.76 and 0.78 for domains physical health, psychological, social relationships and environment, respectively [34].

BSI-18 is a measure of psychological distress widely used in mental health research. This 18-item self-reported questionnaire consists of three domains (depression, anxiety and somatisation). Each domain scores between 0 and 24, while the global scale Global Severity Index (GSI) ranges between 0 and 72 [35]. Higher score represents more psychological distress. Validation of the German version of the instrument has shown good psychometric properties of the GSI with a Cronbach’s alpha of 0.93 and strong correlations with the Patient Health Questionnaire (PHQ) Depression (0.71) and Anxiety (0.73) [36].

GAF is an observer-rated instrument measuring overall level of psychosocial functioning on a scale from 1 to 100 with the higher score indicating better functioning. The instrument is widely used internationally in mental health research and is an integral part of the DSM classification system (Axis V) [37, 38]. Estimates for the test–retest reliability of GAF vary between studies ranging from 0.54 to 0.9 [39]. Moderate correlations with SCL-90-R and weak correlations with the Social Adjustment Scale were reported for GAF [40].

Mini-ICF-APP is an observer-rated instrument of social functioning designed for use by carers of adults with mental disorders [41]. It consists of thirteen domains which are rated on a 0–4 scale where 0 represents ‘no impairment’ and 4 ‘total disability’. Consequently, higher score represents more severe impairment [42]. Test–retest reliability of the instrument in a UK sample was 0.82 and Cronbach’s alpha was 0.86. Strong correlations with the Brief Psychiatric Rating Scale (BPRS) and Social and Occupational Functioning Assessment Scale indicated good validity of the Mini-ICF-APP [41].

The EuroQoL EQ-5D-5L is a generic, self-reported, preference-based questionnaire used to measure HRQoL alongside five domains (mobility, self-care, usual activities, pain/discomfort, depression/anxiety) [43]. The EQ-5D Visual Analogue Scale (VAS) is a measure of current health status where 100 represents full health and 0 represents death. EQ-5D-5L has been validated in the German setting [44]. In a sample of patients with depression, the EQ-5D-5L’s Cronbach’s alpha was 0.77 and correlation with Beck Depression Inventory was − 0.58 showing satisfactory validity [45].

Finally, the OxCAP-MH is a self-reported, 16-item questionnaire of capabilities. Each question is scored on a 1–5 Likert scale, where 1 refers to a very low level of the given capability domain and 5 refers to no limitations in the given capability domain. For easier interpretation, the total OxCAP-MH score which ranges from 16 to 80, is standardised to a 0 to 100 scale, using the formula 100 × (OxCAP-MH total score − minimum score)/range, where 0 represents no capabilities and 100 represents full capabilities [30]. Reliability of the original English OxCAP-MH instrument was assessed based on a sample of 172 patients (Cronbach’s alpha 0.79). Test–retest reliability analysis drawn on sub-sample of 57 patients resulted in an intra-class correlation coefficient (ICC) of 0.86. Assessment of construct validity showed statistically significant positive correlations with the EQ-5D-3L, EQ-5D-VAS and the BPRS.

### Analyses

Data were collected in paper form, entered into the data collection system by the carers and double checked by the data analyst. All data were pseudo-anonymised at source in pro mente kärnten. Questionnaire scores were calculated according to the guidelines provided for the respective measures. Due to the lack of Austrian tariffs for the EQ-5D, German [46] value set was used to calculate utility index (EQ-5D-index). According to the EQ-5D manual, in the absence of a country-specific value set, values of a neighbouring country or population similar to the population under investigation should be used [47]. Therefore, German values were used in the primary analysis. In addition, we run a sensitivity analysis using the UK value set as common practice in countries that do not have their own value sets available [48]. Since the sensitivity analysis results were very similar to those obtained in the primary analysis and the current NICE position statement does not recommend using the current UK 5L valuation set in general [49], we only report results for the German value set. Changes in questionnaire scores between baseline and 6-month follow-up were assessed using paired *t* test.

#### Floor and ceiling effects and item-total correlation

Floor/ceiling effects were considered present when lowest/highest values in the total score were reported by more than 15% of respondents or when more than 40% of respondents scored lowest/highest values on questionnaire items [14, 50]. Item-total correlation is used to confirm the homogeneity of the scale items and it was assessed as the correlation of an individual item with the total score calculated by omitting that item. According to the rule of thumb provided by Streiner et al. correlation between the item and the scale higher than 0.3 indicates satisfactory item homogeneity [51].

#### Exploratory factor analysis (EFA)

EFA is used to study dimensionality of a measurement instrument when a priori assumptions about the dimensional structure of an instrument are lacking [52]. As no prior EFA was performed for the OxCAP-MH questionnaire and no prior hypotheses about the dimensionality of the OxCAP-MH existed, the current study used EFA for two purposes. Firstly, to examine the dimensional structure underlying the OxCAP-MH questionnaire; secondly, to examine whether the OxCAP-MH and EQ-5D-5L items share the same set of the underlying factors or measure separate constructs. Prior to the factor analysis, the Kaiser–Meyer–Olkin (KMO) Measure of Sampling Adequacy and Bartlett’s Test of Sphericity were performed to test for suitability of the data for factor analysis. Good suitability for factor analysis is indicated by a larger KMO (score below 0.5 is considered unacceptable for factor analysis) and statistically significant Bartlett’s Test [53]. Because both of the instruments (OxCAP-MH and EQ-5D-5L) are scored on categorical (polytomous) scales, EFA was performed using polychoric correlations adequate for this type of data [54]. For easier interpretation, we recoded EQ-5D-5L response levels to 1–5 scale, where 1 represents extreme problems and 5 represents no problems (opposite to the traditional scoring of the EQ-5D-5L descriptive system). The number of factors retained was chosen based on criteria which included the Kaiser Criterion, the scree plot and parallel analysis (PA). As a factor extraction method, we used Minimum Rank Factor Analysis (MRFA) recommended by Timmerman and Lorenzo-Seva [54]. In addition, we run EFA with commonly used Unweighted Least Squares as a factor extraction method which yielded similar factor solution as the MRFA. Therefore, we report findings for MRFA only. Factor loadings were rotated using promin rotation [55]. We used the freely available software FACTOR to conduct the EFA [56], for the other analyses we used Stata 15.1 [57].

#### Responsiveness

The anchor-based approach was applied to assess responsiveness. Self-reported quality-of-life measures that correlated with the OxCAP-MH at the minimum level of 0.5 were selected as anchor questionnaires. Correlations were assessed using Spearman’s rank correlation coefficients applying Bonferroni correction to adjust for multiple testing. Correlations of < 0.3, 0.3–0.5 and > 0.5 were considered weak, moderate and strong, respectively [58]. Because of the lack of available data on the value of minimally important difference for the similar study population for the instruments used as anchors, the assumption of ½ standard deviation (SD) of mean baseline score was used as an indicator of a change that is meaningful to patients [59]. If a change in the instrument scores between baseline and 6-month follow-up assessments was estimated beyond ½ SD positively or negatively, patients were classified as “Improved” or “Worsened”, respectively. Otherwise, patients were classified as “No change”. Small, moderate and large magnitude of change was indicated by < 0.5, 0.5–0.79 and ≥ 0.8 values of standardised response mean (SRM), respectively [60]. SRM was calculated as the ratio of the mean change, between baseline and follow-up scores in a single group, to the SD of the change scores [51]. Changes at the individual patient level were also explored reporting percentage of the study respondents who improved, worsened or did not change according to both the OxCAP-MH and the anchor questionnaires.

#### Discriminant validity

Discriminant validity was defined as the ability of the questionnaire to discriminate between subgroups and was assessed by comparing differences in OxCAP-MH scores between defined patient groups based on age, sex, marital and employment status, mental health comorbidity, overall quality-of-life levels and satisfaction with health. Questions about overall QoL and satisfaction with health were derived from the two items of the WHOQOL-BREF instrument which included: “How would you rate your quality of life?” and “How satisfied are you with your health?” [61]. Associations between the OxCAP-MH scores and selected variables were tested using *t* tests for two group comparisons and one-way ANOVA for comparisons between multiple groups. In addition, a multivariable linear regression was used including all patient-related variables as explanatory variables in the model.

#### Test–retest reliability and Cronbach’s alpha

Reliability was defined as the ability of the questionnaire to give consistent results after being administered on multiple occasions to the same person when no changes in health state/wellbeing were reported [62]. Carers were instructed to ask study participants to complete the OxCAP-MH questionnaire at the next scheduled meeting after baseline assessment that was not later than 30 days after the first completion of the questionnaire. Prior to completing the questionnaire, patients were asked: “*Since you last completed this questionnaire, has anything in relation to your health or social situation changed?*” Only patients who gave a negative answer to that question were included in test–retest analysis which was assessed using ICC. ICC estimates of < 0.5, 0.5–0.74, 0.75–0.89 and ≥ 0.9 indicated poor, moderate, good and excellent reliability, respectively [63]. ICC for test–retest analysis was calculated using a two-way random model with absolute agreement [64]. For Cronbach’s alpha, values greater than 0.70 were considered an indicator of good internal consistency [50, 51].

For all analyses, the level of significance was *p* < 0.05. Analyses were conducted on complete cases.

## Results

### Patient characteristics

Out of the typical patient flow of 400 patients who were approached between June 2016–March 2017, 166 patients agreed to participate in the study. Two patients withdrew before completing the baseline assessment. Baseline data were collected for 164 patients, out of whom 159 participants had fully completed OxCAP-MH questionnaires and were included in the baseline analyses. Thirty-one study participants withdrew from the study or were lost to follow-up. One-hundred and twenty seven patients (*n* = 127, 80%) completed fully both the baseline and the 6-month follow-up assessments. No differences in baseline demographic and socio-economic characteristics and questionnaire scores were observed between the patients who completed the study and those who withdrew or were lost to follow-up.

Patient characteristics are presented in Table 1. Mean age was 45 (min. 18, max. 87) and 64% (*n* = 102) of the study sample were women. Participants had a variety of mental health diagnoses with depression (37%) and anxiety disorders (24%) being the most common. A total of 103 (65%) patients had single diagnoses and 45 (28%) patients had multiple mental health diagnoses.

**Table 1:** Patient characteristics

Variable	Baseline (*n* = 159)		6-Month follow-up (*n* = 127)	
	*n*	Mean (SD) or %	*n*	Mean (SD) or %
Age	155	45 (13)	123	45 (13)
Missing	4		4	
Sex				
Men	57	36%	46	36%
Women	102	64%	81	64%
Nationality				
Austrian	147	92%	123	97%
Other	4	2%	2	2%
Missing	8	6%	2	2%
Education				
Primary	2	1%	2	2%
Secondary lower	28	18%	21	16%
Secondary upper	66	41%	54	42%
Tertiary	7	4%	8	6%
Missing	56	35%	42	33%
Source of income				
Social benefits	52	33%	42	33%
Income from employment	30	19%	25	20%
Pension	42	26%	35	28%
Other	10	6%	8	6%
Missing	25	16%	17	13%
Marital status				
Divorced or separated	26	16%	20	16%
Partnership	24	15%	20	16%
Single	51	32%	44	35%
Married	48	30%	38	30%
Widowed	7	4%	5	4%
Missing	3	2%	0	0%
Living situation				
Living with family	21	13%	18	14%
Renting a flat	71	45%	57	45%
Owning a flat	63	40%	50	39%
Missing	4	2%	2	2%
Private insurance				
Yes	11	7%	12	9%
No	148	93%	115	91%
Mental health diagnoses				
Single diagnosis	103	65%		
Multi-morbid diagnoses	45	28%		
Missing	11	7%		
Most common diagnoses^a^				
Organic mental disorders (F00-F09)	15	9%		
Mental disorders due to psychoactive substance use (F10-F19)	12	8%		
Schizophrenia, schizotypal and delusional disorders (F20-F29)	12	8%		
Bipolar disorder (F31)	10	6%		
Depression (F32-F33)	59	37%		
Other mood disorders^b^	2	1%		
Anxiety disorders (F40-F41)	38	24%		
Obsessive–compulsive disorder (F42)	10	6%		
Adjustment disorders (F43)	28	18%		
Other neurotic, stress-related and somatoform disorders^c^	15	9%		
Other	7	4%		
Questionnaire scores				
OxCAP-MH	159	64 (15)	127	67 (16)*
EQ-5D-index	158	0.711 (0.267)	127	0.766 (0.246)*
EQ-5D-VAS	162	61 (22)	132	66 (21)*
BSI-18	152	23 (14)	121	19 (14)*
WHOQOL-BREF Physical	159	56 (20)	126	61 (20)*
WHOQOL-BREF Psychological	159	50 (20)	126	56 (20)*
WHOQOL-BREF Social relationship	159	54 (23)	126	57 (21)*
WHOQOL-BREF Environment	159	67 (15)	126	70 (15)*
Mini-ICF-APP	157	15 (9)	123	12 (8)*
GAF	149	58 (15)	109	62 (15)*

*Statistically significant at 5% level difference between the mean baseline and 6-month follow-up scores based on paired *t* test

^a^The percentages do not add up to 100% because some of the study participants had two or more mental health diagnoses. ICD-10 codes for the diagnoses are provided in parentheses

^b^F30-F39, without F31-F33

^c^F40-F48, without F40-F43

### Analyses

#### Floor and ceiling effects and item-total correlation

**Fig. 1:**
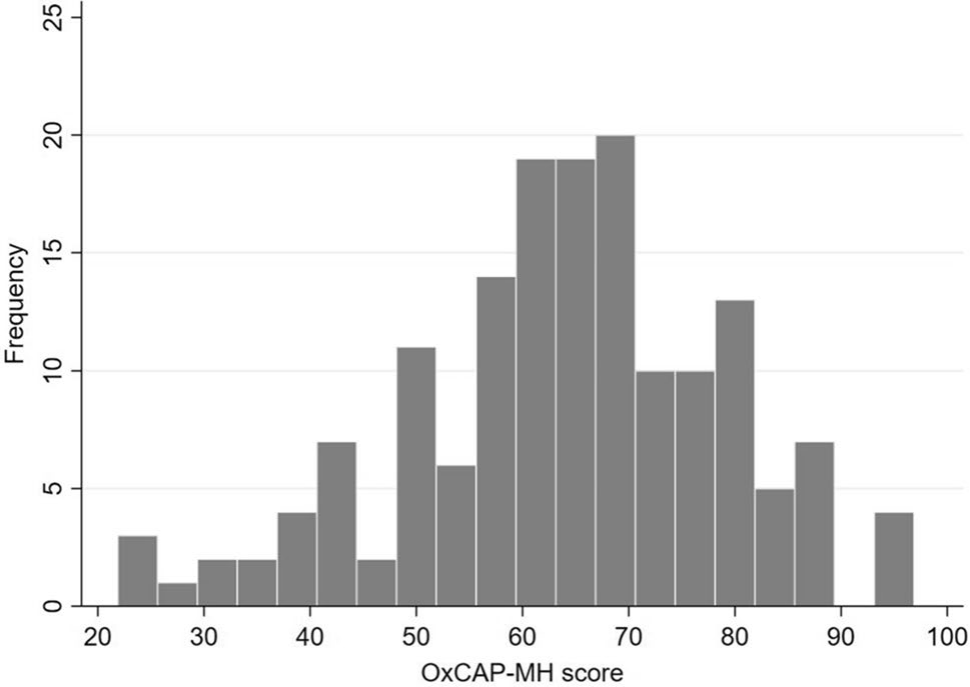
Distribution of the baseline OxCAP-MH total score

The baseline OxCAP-MH scores were normally distributed (Fig. 1) showing no floor/ceiling effects in the overall OxCAP-MH score. Ceiling effects were observed in the items ‘appreciating nature’ (64%), ‘probability of assault’ (48%) and ‘respecting people around’ (43%), indicating no limitations in these capability domains for the majority of the patients. Item-total correlations of the individual items were satisfactory and ranged between 0.29 and 0.61 (Online Supplement Table 2).

#### Exploratory factor analysis (EFA)

Kaiser’s measure (KMO) was 0.835 for the OxCAP-MH items alone and 0.857 for pooled OxCAP-MH and EQ-5D items and the p-value for Bartlett’s Test was < 0.001, both representing adequacy of the data for factor analysis. Investigation of the Kaiser Criterion, scree plot and PA suggested that a two-factor solution was most suitable for the OxCAP-MH alone as well as for the pooled OxCAP-MH and EQ-5D-5L questionnaire items (Online Supplement Figs. 1 and 2). Nine OxCAP-MH domains loaded on Factor 1 (Table 2). These domains refer to contextual aspects of one’s life that include personal and environmental dimensions (‘suitable flat situation’, ‘local decisions’, ‘appreciation of nature’, ‘respect for people around’, ‘enjoy love and support’, ‘freedom of deciding for yourself’, ‘creativity‘, ‘freedom of expression’, ‘access to interesting activities’). We refer to this factor as the ‘contextual factor’. The remaining seven OxCAP-MH domains loaded on Factor 2. These items are related to limitations in one’s health (‘limit daily activities’, ‘less sleep over worrying’, ‘free time activities’) and social sphere (‘neighbourhood safety’, probability of discrimination’, ‘probability of assault’, ‘meet socially with friends or family’). Hence, we refer to this factor as the ‘sociophysical factor’.

**Table 2:** Exploratory factor analysis (EFA) of the OxCAP-MH items using promin rotation (*n* = 159)

Variable	Rotated factor loadings		OxCAP-MH dimensions	Central human capabilities (Nussbaum [11])
	Factor 1	Factor 2		
T0_OXCAP1		0.66	Limit daily activities	Bodily health
T0_OXCAP2		0.70	Meet socially with friends or family	Affiliation
T0_OXCAP3		0.71	Less sleep over worries	Emotions
T0_OXCAP4		0.52	Enjoy free time activities	Play
T0_OXCAP5	0.36		Suitable flat situation	Bodily health
T0_OXCAP6		0.71	Safety in neighbourhood	Bodily integrity
T0_OXCAP7		0.58	Probability of assault	Bodily integrity
T0_OXCAP8		0.61	Probability of discrimination	Affiliation
T0_OXCAP9a	0.51		Local decisions	Control over one’s environment
T0_OXCAP9b	0.37		Freedom of expression	Senses, imagination & thought
T0_OXCAP9c	0.91		Appreciation of nature	Species
T0_OXCAP9d	0.88		Respect for people around	Affiliation
T0_OXCAP9e	0.53		Enjoy love and support	Emotions
T0_OXCAP9f	0.58		Freedom of deciding for yourself	Practical reason
T0_OXCAP9g	0.78		Creativity	Senses, imagination & thought
T0_OXCAP9h	0.64		Access to interesting activities/employment	Control over one’s environment
Cronbach’s alpha	0.82	0.76		

Loadings < 0.2 were removed; correlation between the factors was 0.51

Two-factor solution was also optimal for the EFA for pooled OxCAP-MH and EQ-5D-5L questionnaire items (Table 3). All five EQ-5D domains strongly loaded on Factor 2 together with the seven OxCAP-MH domains covered by defined earlier ‘sociophysical factor’. The remaining OxCAP-MH domains loaded on Factor 1 earlier defined as ‘contextual factor’.

**Table 3:** Exploratory factor analysis (EFA) of the OxCAP-MH and EQ-5D-5L items using promin rotation (*n* = 154)

Variable	Domain	Rotated factor loadings	
		Factor 1	Factor 2
OXCAP1	Limit daily activities		0.60
OXCAP2	Meet socially with friends or family		0.61
OXCAP3	Less sleep over worries		0.54
OXCAP4	Enjoy free time activities		0.57
OXCAP5	Suitable flat situation	0.38	
OXCAP6	Safety in neighbourhood		0.53
OXCAP7	Probability of assault		0.24
OXCAP8	Probability of discrimination		0.47
OXCAP9a	Local decisions	0.39	
OXCAP9b	Freedom of expression	0.44	
OXCAP9c	Appreciation of nature	0.74	
OXCAP9d	Respect for people around	0.89	
OXCAP9e	Enjoy love and support	0.52	
OXCAP9f	Freedom of deciding for yourself	0.54	
OXCAP9g	Creativity	0.75	
OXCAP9h	Access to interesting activities/employment	0.58	
EQ-5D-5L 1	Mobility		0.88
EQ-5D-5L 2	Self-care		0.90
EQ-5D-5L 3	Daily activities		0.77
EQ-5D-5L 4	Pain/discomfort		0.69
EQ-5D-5L 5	Depression/anxiety		0.68
Cronbach’s alpha		0.87	0.80

Note: loadings < 0.2 were removed; Correlation between the factors was 0.37

#### Responsiveness

**Fig. 2:**
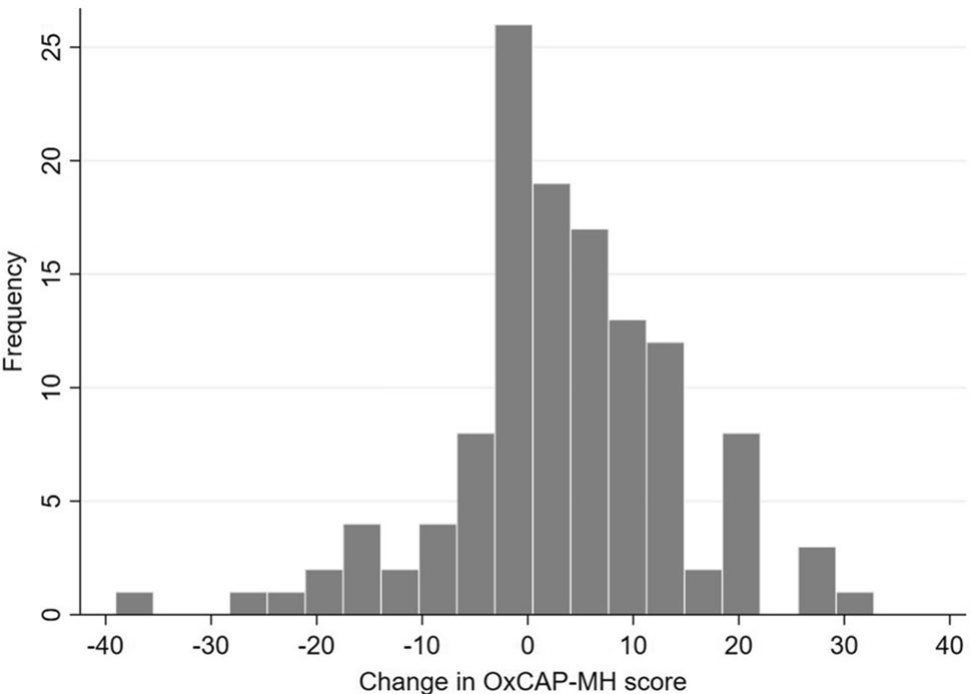
Distribution of the change in OxCAP-MH score between baseline and 6-month follow-up

Overall, study participants significantly improved in all questionnaire scores between baseline and 6-month follow-up (Table 1). Distribution of change in OxCAP-MH scores shows that majority of participants improved (*n* = 75, 60%) or did not change (*n* = 16, 13%), and around one-quarter of the participants worsened (*n* = 33, 27%) (Fig. 2).

Correlation coefficients of the baseline and follow-up scores were strong between the OxCAP-MH and EQ-5D-index, EQ-5D-VAS, BSI-18, WHOQOL-BREF Physical, WHOQOL-BREF Psychological, WHOQOL-BREF Environment (Table 4). Moderate correlations of the baseline and follow-up scores were observed for WHOQOL-BREF Social relationships, Mini-ICF-APP and GAF. Correlations of change scores between OxCAP-MH and other measures were weak to moderate and ranged from − 0.10 (Mini-ICF-APP) to 0.51 (WHOQOL-BREF Psychological)

**Table 4:** Correlation coefficients of OxCAP-MH with other measures (Spearman’s correlations)

	OxCAP-MH		
	Baseline	Follow-up	Change score
EQ-5D-index	**0.66**	**0.64**	**0.30**
EQ-5D-VAS	**0.58**	**0.56**	**0.31**
BSI-18	**− 0.67**	**− 0.72**	**− 0.42**
WHOQOL-BREF physical health	**0.69**	**0.71**	**0.43**
WHOQOL-BREF psychological	**0.75**	**0.75**	**0.51**
WHOQOL-BREF social relationships	**0.50**	**0.48**	0.19
WHOQOL-BREF environment	**0.69**	**0.70**	**0.31**
Mini-ICF-APP	**− 0.47**	**− 0.41**	**− **0.10
GAF	**0.35**	**0.36**	0.15

Correlation coefficients significant at the 5% level are marked bold; Bonferroni adjustment was applied to calculated significance levels

Based on correlation analyses, EQ-5D-index, EQ-5D-VAS, BSI-18 and WHOQOL-BREF Physical, Psychological and Environment were chosen as anchor questionnaires. For the study participants who reported improvement in BSI-18 and WHOQOL-BREF Physical and Psychological scores, the improvement in the OxCAP-MH score was statistically significant at the 1% level with large SRM statistics. Statistically significant improvements in the OxCAP-MH scores at the 1% level with moderate SRM statistics were observed for those who improved in EQ-5D-index, EQ-5D-VAS and WHOQOL-BREF Environment scores (Table 5).

**Table 5:** Responsiveness of the OxCAP-MH to changes in health status defined as ½ SD change from mean baseline questionnaire scores

Instrument (No. of complete cases)	Change in instruments scores^a^	*n*	OxCAP-MH score				
			Mean (SD) baseline	Mean (SD) follow-up	Mean change (SD)	*p* value^b^	SRM*
EQ-5D-index (*n* = 116)	Improved	33	54.31 (15.69)	61.55 (15.84)	7.24 (11.70)	**<0.001**	*0.62*
	Worsened	12	64.45 (15.08)	61.72 (18.59)	− 2.73 (8.46)	0.29	− 0.32
	No change	71	69.05 (13.65)	70.91 (15.71)	1.85 (10.53)	0.14	0.17
EQ-5D-VAS (*n* = 122)	Improved	36	57.99 (14.81)	65.28 (15.03)	7.29 (11.24)	**<0.001**	*0.65*
	Worsened	17	67.28 (15.76)	63.14 (17.94)	− 4.14 (13.69)	0.23	− 0.30
	No change	69	66.28 (15.31)	69.56 (15.42)	3.28 (8.70)	**<0.001**	0.38
BSI-18 (*n* = 108)	Improved	29	56.41 (14.47)	65.52 (13.67)	9.10 (10.82)	**<0.001**	**0.90**
	Worsened	17	65.07 (9.23)	64.98 (12.77)	− 0.09 (8.16)	0.96	− 0.01
	No change	62	67.06 (17.04)	68.22 (18.95)	1.16 (11.49)	0.43	0.13
WHOQOL-BREF physical health (*n* = 117)	Improved	46	59.82 (17.34)	69.19 (16.18)	9.37 (10.06)	**<0.001**	**0.93**
	Worsened	16	68.36 (13.37)	62.69 (17.17)	− 5.66 (13.24)	0.11	− 0.43
	No change	55	65.88 (14.85)	66.53 (16.67)	0.65 (8.72)	0.58	0.07
WHOQOL-BREF psychological (*n* = 117)	Improved	46	61.00 (17.67)	69.80 (17.02)	8.80 (10.38)	**<0.001**	**0.85**
	Worsened	15	66.25 (14.20)	60.42 (16.05)	− 5.83 (13.96)	0.13	− 0.42
	No change	56	65.51 (14.73)	66.57 (15.99)	1.06 (8.70)	0.37	0.12
WHOQOL-BREF environment (*n* = 117)	Improved	42	62.09 (16.52)	69.01 (15.83)	6.92 (10.92)	**<0.001**	*0.63*
	Worsened	16	60.25 (12.86)	55.96 (18.26)	− 4.30 (14.28)	0.25	− 0.30
	No change	59	66.05 (16.15)	68.67 (15.63)	2.62 (9.47)	**0.04**	0.28

*Values < 0.5, 0.5–0.79 and ≥ 0.8 represent small (in normal value), moderate (in italic value) and large (in bold value) SRM statistics, respectively

^a^Changes in instrument scores between baseline and 6-month follow-up were categorised as improved, worsened or no change, based on a change of 0.5 standard deviation of the mean baseline assessment

^b^Paired *t* test

For the individual patient-level changes in the questionnaire scores, 41% and 42% of patients who improved in WHOQOL-BREF Environment and EQ-5D-index, respectively, improved also in the OxCAP-MH score, while 54% of those who improved according to the WHOQOL-BREF Physical and Psychological health, also improved in the OxCAP-MH score (Table 6). Of those who did not change according to the BSI-18, EQ-5D-index, WHOQOL-BREF Environment, EQ-5D-VAS or WHOQOL-BREF Physical and Psychological health scores, 56, 59, 61, 62, 64 and 70% also did not change based on the OxCAP-MH score, respectively.

**Table 6:** Responsiveness of the OxCAP-MH to changes in health status defined as ½ SD change from mean baseline questionnaire scores

Instrument (no. of complete cases)	Change in instruments scores^a^	*n*	Change in OxCAP-MH score^a^		
			Improved	Worsened	No change
EQ-5D-index (*n* = 116)	Improved	33	14 (42%)	4 (12%)	15 (45%)
	Worsened	12	1 (8%)	2 (17%)	9 (75%)
	No change	71	20 (28%)	9 (13%)	42 (59%)
EQ-5D-VAS (*n* = 122)	Improved	36	16 (44%)	3 (8%)	17 (47%)
	Worsened	17	3 (18%)	4 (23%)	10 (59%)
	No change	69	19 (28%)	7 (10%)	43 (62%)
BSI-18 (*n* = 108)	Improved	29	14 (48%)	2 (7%)	13 (45%)
	Worsened	17	3 (18%)	2 (12%)	12 (70%)
	No change	62	17 (27%)	10 (16%)	35 (56%)
WHOQOL-BREF physical (*n* = 117)	Improved	46	25 (54%)	1 (2%)	20 (44%)
	Worsened	16	1 (6%)	5 (31%)	10 (63%)
	No change	55	11 (20%)	9 (16%)	35 (64%)
WHOQOL-BREF psychological (*n* = 117)	Improved	46	25 (54%)	3 (7%)	18 (39%)
	Worsened	15	2 (13%)	5 (33%)	8 (54%)
	No change	56	10 (18%)	7 (12%)	39 (70%)
WHOQOL-BREF environment (*n* = 117)	Improved	42	17 (41%)	3 (7%)	22 (52%)
	Worsened	16	4 (25%)	5 (31%)	7 (44%)
	No change	59	16 (27%)	7 (12%)	36 (61%)

^a^Changes in instrument scores between baseline and 6-month follow-up were categorised as improved, worsened or no change, based on a change of 0.5 standard deviation of the mean baseline assessment

#### Discriminant validity

In the univariable analysis, OxCAP-MH significantly discriminated between employed and unemployed participants, between single-morbid and multi-morbid respondents and between different levels of subjective overall quality of life and satisfaction with health. In the multivariable regression framework, statistically significant associations were maintained for multi-morbidity, overall quality of life and satisfaction with health (Online Supplement Table 3). No statistically significant associations with the variables sex, age and marital status were confirmed.

#### Test–retest reliability and Cronbach’s alpha

For the test–retest analysis, a total of 146 patients (92%) completed the questionnaire a second time before the follow-up assessment. Forty-three cases were excluded due to indication of changed health state during the observation period. A further 34 questionnaires were excluded from the analysis due to the a priori set maximum 30 day timeframe limitation of this test. Overall, 69 patients could be included in the ‘0–30 days’ test–retest analysis. The estimated single-measure intra-class correlation was 0.80 (95%CI 0.69–0.87) indicating good similarity between two assessments within individual respondents. Sensitivity analyses were performed for 21 (*n* = 39) and 14 (*n* = 28) days’ time spans. Details are presented in Table 7. Cronbach’s alpha for the OxCAP-MH questionnaire was 0.85.

**Table 7:** Results of the test–retest analysis

Time between assessments (days)	No. of participants	Single-measure intra-class correlation	Linear regression (coefficient, *p* value and *R*^2^)
0–30	69	0.80 (95% CI 0.69–0.87)	0.80, *p* < 0.001, *R*^2^ = 0.68
0–21	39	0.83 (95% CI 0.70–0.91)	0.85, *p* < 0.001, *R*^2^ = 0.73
0–14	28	0.86 (95% CI 0.72–0.92)	0.96, *p* < 0.001, *R*^2^ = 0.80

## Discussion

This study examined the German language version of the OxCAP-MH, a multi-dimensional capability instrument for outcome measurement in mental health research. The original English version of this instrument was developed in the UK and validated for severe mental disorders in the English setting showing robust psychometric properties [7, 14]. After careful linguistic translation and cultural validation process [29], the German language version of the OxCAP-MH was tested and fully validated in Austria. In addition, the broader concept of capabilities in comparison to HRQoL was formally investigated through comparison with EQ-5D-5L in an EFA.

The German language version of the OxCAP-MH proved to be a measure of quality of life and broader wellbeing beyond traditional HRQoL assessed by the EQ-5D-5L. In the EFA, all EQ-5D-5L domains loaded strongly on the so-called sociophysical factor while the remaining OxCAP-MH items loaded on the separate second factor that represents capability dimensions beyond health. These results indicate that in comparison to the EQ-5D-5L, the OxCAP-MH may be seen as enhanced rather than complementary in its concept. This finding is different from those of the two studies that looked into the factor structure of the ICECAP-A and ICECAP-O in comparison to the EQ-5D-3L. These studies showed that these questionnaires measure two distinct concepts and should be considered complementary measures since they provide different information about the patient’s wellbeing [21, 65].

Moderate to strong level of convergence of the baseline and follow-up scores between OxCAP-MH and other measures, including EQ-5D-index, EQ-5D-VAS, four dimensions of WHOQOL-BREF, BSI-18 and Mini-ICF-APP, confirmed that different domains of quality of life such as social functioning, health status and psychological distress are well reflected in the OxCAP-MH score. Similar to the UK validation results, the weakest correlation was reported between the OxCAP-MH and the GAF. This was expected also in the current context, as the GAF score reflects a clinician’s view on patient’s symptoms, impairment and functioning, and not the patient’s own perceived wellbeing.

The current validation study also investigated new aspects of the OxCAP-MH instrument including its responsiveness and discriminant validity. These analyses showed that the OxCAP-MH is responsive to the improvements in a patient’s health state over time measured by anchor questionnaires and it discriminates among defined patient groups with high sensitivity. Only few studies examined capability instruments’ responsiveness over time [20, 66, 67]. Furthermore, there are relatively few studies that looked at the use of capability measures among patients suffering from mental disorders [20, 68, 69], and these emphasised good construct validity and feasibility of the instruments. Given the existing literature, this study is unique in providing a full psychometric validation of a capability measure for a cohort with mixed mental health problems and its comparison to generic HRQoL.

As opposed to the validation of the English version, in this study, data collection took place in the real-world care setting and confirmed both the feasibility and validity of using the OxCAP-MH in the routine evaluation of mental health services. Our findings that the OxCAP-MH is versatile across different mental health diagnoses and can be used both in clinical and health economic research in diverse settings are in-line with preliminary evidence from other ongoing studies that utilise the instrument [26–28, 70].

Similar to the UK validation study of the English version with patients suffering from psychosis, the German language OxCAP-MH had good internal consistency with a Cronbach’s alpha of 0.85 and showed good reliability in test–retest analysis with ICC estimates of 0.80. These findings are similar to other studies on capability measures. For example, Rand et al. reported ICC of 0.78 for the ASCOT-SCT4 measure [69] and Al-Janabi et al. reported ICC of 0.72 for the ICECAP-A index score [71].

Despite limitations by its pragmatic design, including limited sample size, some degree of missing data on the diagnoses and socio-economic status and limited possibilities of conducting test–retest analysis in strictly defined time period, this study confirms the favourable psychometric properties of the instrument also in diverse cultural and disease settings. The study provides also new evidence on the responsiveness of the OxCAP-MH and its conceptual ability to capture broader aspects of wellbeing beyond the traditional HRQoL framework. The findings further support the use of the OxCAP-MH as an outcome measure both in experimental and in routine evaluation of health and social care interventions also the in German-speaking context.

## Electronic supplementary material

Below is the link to the electronic supplementary material.

Supplementary material 1 (DOCX 130 kb)
