# Supplementary material for: Measuring broader wellbeing in mental health services: validity of the German language OxCAP-MH capability instrument
Source: Qual Life Res. 2019 Apr 27;28(8):2311–23. doi: 10.1007/s11136-019-02187-9 (PMC6620251; doi:10.1007/s11136-019-02187-9)
Supplement: Supplementary file 1 — Supplementary material 1 (DOCX 130 kb) [file 11136_2019_2187_MOESM1_ESM.docx]

**Online Supplement**

**Title:** Measuring broader wellbeing in mental health services: validity of the German language OxCAP-MH capability instrument

**Journal name:** Quality of Life Research

**Authors:** Agata Łaszewska^1^, Markus Schwab^2^, Eva Leutner^3^, Marold Oberrauter^3^, Georg Spiel^2,3^, Judit Simon^1,4,5*^

**Affiliations:**

^1^ Department of Health Economics, Center for Public Health, Medical University of Vienna, Kinderspitalgasse 15/1, 1090 Vienna, Austria

^2^ pro mente Forschung, Villacher Straße 161, 9020 Klagenfurt am Wörthersee, Austria

^3^ pro mente kärnten GmbH, Villacher Straße 161, 9020 Klagenfurt am Wörthersee, Austria

^4^ Department of Psychiatry, University of Oxford, Warneford Hospital, Warneford Ln, Oxford OX3 7JX, UK

^5^ HERC, Nuffield Department of Population Health, University of Oxford, Richard Doll Building, Old Road Campus, Oxford OX3 7LF, UK

***Corresponding author:** Judit Simon, Department of Health Economics, Center for Public Health, Medical University of Vienna, Kinderspitalgasse 15/1, 1090 Vienna; e-mail: [judit.simon@meduniwien.ac.at](mailto:judit.simon@meduniwien.ac.at); tel: +43 1 40160 – 34841, fax: +43 (0)1 40160 934840

**Table 1. Nussbaum's ten central human capabilities and related OxCAP-MH questionnaire items**

| Central human capabilities (Nussbaum, 2000) | Explanation (Nussbaum, 2000) | OxCAP-MH dimensions  (number of the questionnaire item) |
| --- | --- | --- |
| Life expectancy^1^ | Being able to live to the end of human life of normal length | - |
| Bodily health | Being able to have a good health including reproductive health, to be adequately nourished, to have adequate shelter | limit daily activities (1);  suitable flat situation (5) |
| Emotions | Being able to have attachments to things and people outside ourselves | less sleep over worries (3);  enjoy love and support (9e) |
| Play | Being able to laugh, to play, to enjoy recreational activities | enjoy free time activities (4) |
| Bodily integrity | Being able to move freely from place to place; having one’s bodily boundaries treated as sovereign | safety in neighbourhood (6);  probability of assault (7) |
| Affiliation | Being able to live with and toward others, to recognize and show concern for other human beings. Having social bases of self-respect and non-humiliation; protection against discrimination | probability of discrimination (8); respect for people around (9d); meet socially with friends or family (2) |
| Control over one’s environment | A: Political. Being able to participate effectively in political choices  B: Material. Having rights to seek employment on an equal basis with others | local decisions (9a);  access to interesting  activities/employment (9h) |
| Senses, imagination & thought | Being able to use the senses, to imagine, think and reason; freedom of expression | creativity (9g);  freedom of expression (9b) |
| Other species | Being able to live with concern for and in relation to animals, plants, and the world of nature | appreciation of nature (9c) |
| Practical reason | Being able to form a conception of the good and to engage in critical reflection about the planning of one’s life | freedom of deciding for yourself (9f) |

Note: ^1^ In the validation study of the English version of the OxCAP-MH, a questionnaire item related to the domain ‘life expectancy’ has been removed due to its limitations and has not been replaced; therefore, this capability domain is not represented in the OxCAP-MH (Simon et al., 2013).

**Table 2. Mean, standard deviation and item-total correlation of the OxCAP-MH items (n=159)**

| Questionnaire’s item | Capability dimension | Mean | Std. Dev. | Item-total correlation^1^ | Ceiling effects^2^ | Floor effects^3^ |
| --- | --- | --- | --- | --- | --- | --- |
| OXCAP1 | limit daily activities | 2.91 | 1.18 | 0.41 | 13% | 11% |
| OXCAP2 | meet socially with friends or family | 3.40 | 1.08 | 0.45 | 16% | 4% |
| OXCAP3 | less sleep over worries | 2.79 | 1.11 | 0.45 | 9% | 13% |
| OXCAP4 | enjoy free time activities | 2.93 | 1.11 | 0.44 | 6% | 11% |
| OXCAP5 | suitable flat situation | 3.87 | 1.04 | 0.42 | 30% | 3% |
| OXCAP6 | safety in neighbourhood | 3.66 | 1.16 | 0.47 | 29% | 4% |
| OXCAP7 | probability of assault | 4.08 | 1.05 | 0.29 | **48%** | 1% |
| OXCAP8 | probability of discrimination | 3.52 | 1.23 | 0.50 | 28% | 4% |
| OXCAP9a | local decisions | 2.75 | 1.29 | 0.29 | 9% | 21% |
| OXCAP9b | freedom of expression | 3.86 | 1.06 | 0.37 | 30% | 4% |
| OXCAP9c | appreciation of nature | 4.57 | 0.67 | 0.44 | **64%** | 1% |
| OXCAP9d | respect for people around | 4.28 | 0.74 | 0.55 | **43%** | 0% |
| OXCAP9e | enjoy love and support | 3.59 | 1.17 | 0.56 | 24% | 4% |
| OXCAP9f | freedom of deciding for yourself | 3.75 | 1.18 | 0.61 | 32% | 6% |
| OXCAP9g | creativity | 3.64 | 1.20 | 0.49 | 27% | 7% |
| OXCAP9h | access to interesting activities/employment | 3.22 | 1.35 | 0.57 | 21% | 15% |

Note: ^1^ Item-total correlation - correlation of the individual item with the scale total omitting that item; ^2^ Ceiling effects are observed when 40% or more respondents reported the highest value on a specific questionnaire item; ^3^ Floor effects are observed when 40% or more respondents reported the lowest value on a specific questionnaire item


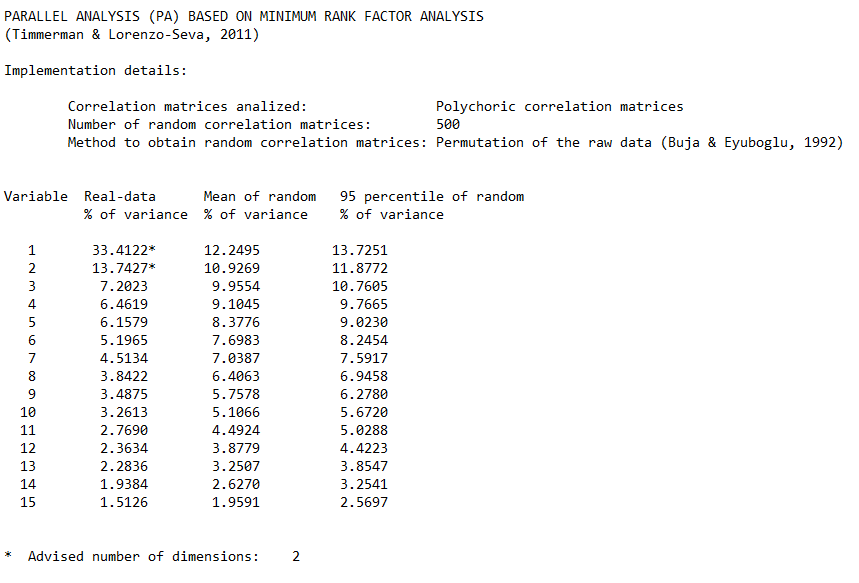


**Figure 1. Parallel analysis (PA) for pooled OxCAP-MH and EQ-5D-5L questionnaire items produced in programme FACTOR**

Note: Parallel analysis (PA) compares eigenvalues generated from the randomly generated raw data matrix against the eigenvalues from the real dataset. A factor is extracted when the eigenvalue from the real dataset exceeds the eigenvalue from the randomly generated data (Thompson & Daniel, 1996). Based on the above PA, the advised number of factors to extract was 2.


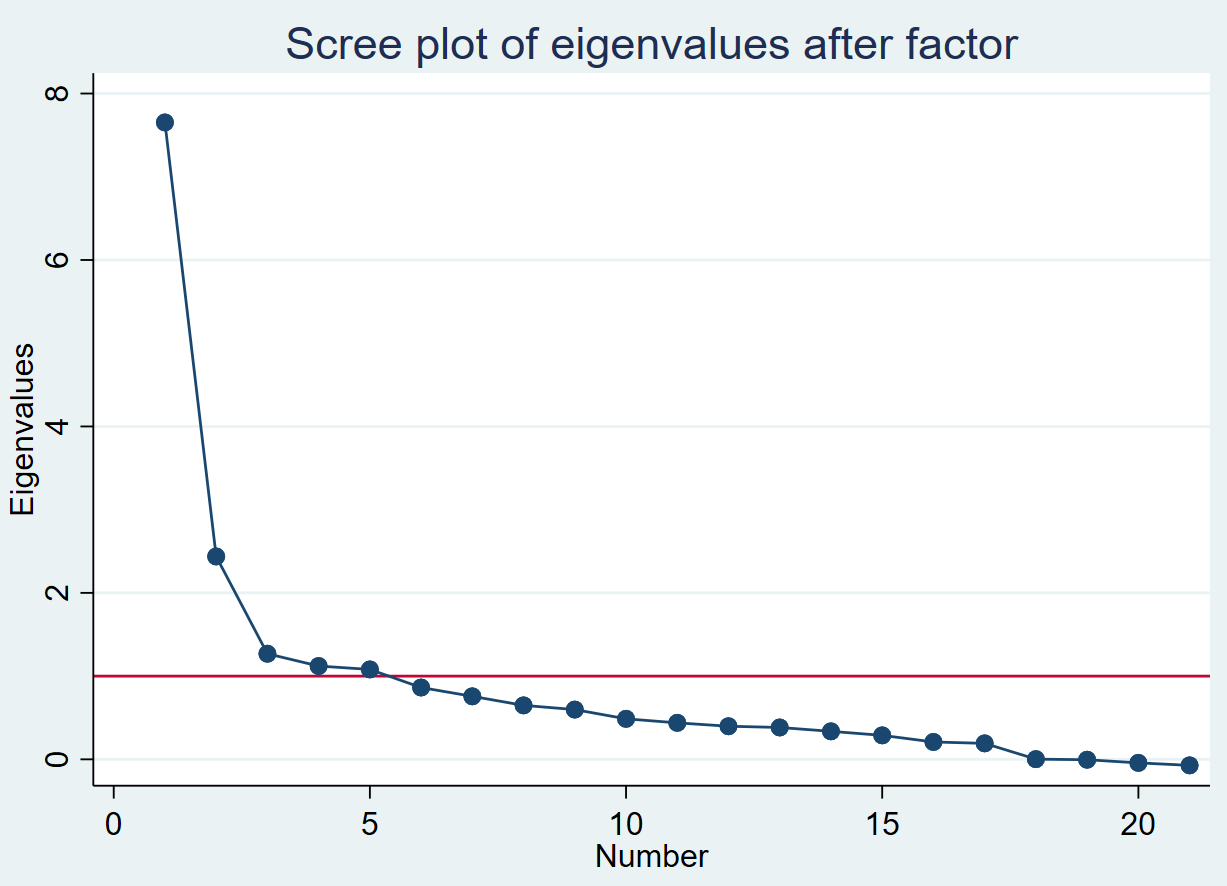


**Figure 2. Scree plot produced in Stata v.15**

Note: Scree plot is a graphical representation of ploted eigenvalues for the respective factors. The rule of thumb is to retain factors that are above the so-called scree, where the plot line starts to level off.

The Scree plot was created for the factor analysis for pooled OxCAP-MH and EQ-5D-5D items which was carried out on a matrix of polychromic correlations, following command *polychoric*, in Stata. Scree plot shows that, although five factors meet Kaiser Criterion (factors with eigenvalue > 1), two factors are above the flattened line which would suggest these two factors should be retained.

**Table 3. Univariable and multivariable associations between the OxCAP-MH score and patient’s characteristics**

|  |  | OxCAP-MH score | | | Univariable analysis | | Multivariable analysis | |
| --- | --- | --- | --- | --- | --- | --- | --- | --- |
| Variable | Level | n | mean | SD | coef. | p-value | coef. | p-value |
| Age | ≤50 | 91 | 65.16 | 15.82 |  |  |  |  |
|  | >50 | 64 | 62.37 | 14.14 | -2.78 | 0.26 | -1.46 | 0.60 |
| Sex | Male | 57 | 61.65 | 15.91 |  |  |  |  |
|  | Female | 102 | 64.98 | 14.48 | 3.33 | 0.18 | -1.17 | 0.64 |
| Employment | No^a^ | 104 | 61.08 | 14.30 |  |  |  |  |
|  | Yes | 30 | 72.86 | 13.62 | 11.77 | **<0.001** | 3.82 | 0.32 |
| Marital status | Single^b^ | 84 | 62.10 | 15.07 |  |  |  |  |
|  | Married or in partnership | 72 | 65.77 | 15.16 | 3.67 | 0.13 | 0.20 | 0.94 |
| Multi-morbidity | one Axis I diagnosis^c^ | 60 | 68.20 | 14.35 |  |  |  |  |
|  | ≥2 Axis I diagnoses | 45 | 56.01 | 16.76 | -12.20 | **<0.001** | -6.55 | **0.01** |
| How would you rate your quality of life? | Very poor or poor | 28 | 48.04 | 15.38 |  |  |  |  |
|  | Neither poor nor good | 75 | 62.25 | 11.52 | 14.20 | **<0.001** | 13.96 | **<0.001** |
|  | Good or very good | 52 | 74.30 | 11.20 | 26.26 | **<0.001** | 23.46 | **<0.001** |
| How satisfied are you with your health? | Very dissatisfied or dissatisfied | 60 | 56.17 | 14.35 |  |  |  |  |
|  | Neither satisfied nor dissatisfied | 44 | 62.42 | 14.30 | 6.26 | **0.02** | 1.02 | 0.77 |
|  | Satisfied or very satisfied | 51 | 73.74 | 10.88 | 17.57 | **<0.001** | 7.28 | **0.03** |

Note: ^a^social benefits as source of income, retired or on pension, or other source of income; ^b^divorced, separated, single, widowed; ^c^Axis I diagnoses include diseases coded in ICD-10 as F00-F99 except for personality disorder F60-F69

**References**

Nussbaum MC (2000) Women and human development: The capabilities approach. Cambridge University Press

Simon J, Anand P, Gray A, Rugkasa J, Yeeles K, Burns T (2013) Operationalising the capability approach for outcome measurement in mental health research. Soc Sci Med 98:187-196.

Thompson, B., & Daniel, L.G. (1996). Factor analytic evidence for the construct validity of scores: A historical overview and some guidelines. Sage Publications Sage CA: Thousand Oaks, CA.
